# Dynamic Multi-LiDAR Based Multiple Object Detection and Tracking

**DOI:** 10.3390/s19061474

**Published:** 2019-03-26

**Authors:** Muhammad Sualeh, Gon-Woo Kim

**Affiliations:** Intelligent Robotics Laboratory, Department of Control and Robot Engineering, Chungbuk National University, Chungdae-ro 1, Seowon-Gu, Cheongju, Chungbuk 28644, Korea; er.sualeh@gmail.com

**Keywords:** data association, ground classification, multiple object detection, multiple object tracking

## Abstract

Environmental perception plays an essential role in autonomous driving tasks and demands robustness in cluttered dynamic environments such as complex urban scenarios. In this paper, a robust Multiple Object Detection and Tracking (MODT) algorithm for a non-stationary base is presented, using multiple 3D LiDARs for perception. The merged LiDAR data is treated with an efficient MODT framework, considering the limitations of the vehicle-embedded computing environment. The ground classification is obtained through a grid-based method while considering a non-planar ground. Furthermore, unlike prior works, 3D grid-based clustering technique is developed to detect objects under elevated structures. The centroid measurements obtained from the object detection are tracked using Interactive Multiple Model-Unscented Kalman Filter-Joint Probabilistic Data Association Filter (IMM-UKF-JPDAF). IMM captures different motion patterns, UKF handles the nonlinearities of motion models, and JPDAF associates the measurements in the presence of clutter. The proposed algorithm is implemented on two slightly dissimilar platforms, giving real-time performance on embedded computers. The performance evaluation metrics by MOT16 and ground truths provided by KITTI Datasets are used for evaluations and comparison with the state-of-the-art. The experimentation on platforms and comparisons with state-of-the-art techniques suggest that the proposed framework is a feasible solution for MODT tasks.

## 1. Introduction

Automated driving systems capable of performing all driving tasks under all roadway and environmental conditions manageable by a human counterpart, is classified as the highest level of automation by the Society of Automotive Engineers (SAE) International [1]. Although Advanced Driving Assistants (ADAs) are commercially available, they either require human intervention or operate only under specific environmental conditions. The realization of the said autonomy has put forth huge requirements on the associated research domains, including Multiple Object Detection and Tracking (MODT). Understanding the dynamic properties of coexisting entities in the environment is crucial to enhance the overall automation, as it directly impacts the quality of localization, mapping, and motion planning [2]. Over the past decade numerous MODT approaches have been studied, traditionally using cameras for perception. A detailed review on the topic is provided in [3]. Objects are detected in the camera reference frame either in a 2D coordinate system, or in a 3D coordinate system under a stereo setup, producing 2D or 3D trajectories, respectively. However, the spatial information is obtained by utilizing camera geometry with inconsistent accuracy, whereas the field of view (FOV) remains limited. Furthermore, panoramic camera-based tracking is yet to be investigated. The camera-based approaches also face inherent challenges, including object truncation, poor lighting conditions, high speed targets, sensor motion, and interactions between targets. The alternate technology of Light Detector and Ranging (LiDAR) that provides sparse panoramic information of the environment has become increasingly popular [4]. With LiDARs being capable of providing panoramic sparse measurements, ranging up to 100 m, and at a reasonable rate of 10–15 Hz, they are the ideal sensors for MODT tasks.

The prominent LiDAR-based MODT approaches follow tracking by detection methods. In this method, a set of trackable objects is identified by a detector. Subsequently, the detected set of objects is processed for pose and centroid estimation, accompanied by a known model fitting. This generates a valid measurement of an object which attributes further evolve over time. These measurements are fed to the state estimation filter to predict the kinematic states of the objects. The optimal Bayesian filters are utilized to estimate the possible evolution of object states in the presence of uncertainties. Later, a data association process is done to assign the detected object to either an existing track or initiation of a new track. To ensure that the unique identities of detected objects are maintained in the cluttered environment, Bayesian probabilistic approaches are deployed. Finally, a track management module is used to heuristically maintain the tracks and to cancel out the spurious ones. However, the most challenging part in LiDAR-based MODT frameworks is efficient object detection and classification. Furthermore, practical implementation of MODT requires real-time performance in cluttered urban environments, using limited computational resources.

In this work, a complete MODT framework is proposed that relies on multiple LiDARs for perception. The detection component involves the slope-based ground removal of LiDAR point clouds, and a 3D grid-based clustering technique for segmentation and classification of objects. This enables the detection of trackable objects under elevated structures. Whereas the tracking component operates on Joint Probabilistic Data Association Filter (JPDAF) coupled with Interacting Multiple Model (IMM), and Unscented Kalman Filter (UKF). Furthermore, the inherent shortcomings of JPDAF pertaining to the combinatorial explosion problem [5] is also handled. This work is a part of smart car project based on a V2X system, involving two platforms with slightly dissimilar configuration and placement of LiDAR sensors. To the best of authors’ knowledge, the proposed MODT framework is the first to have been implemented on an embedded computer that is based on multiple LiDARs, performs in real-time, and considers the existence of non-planar ground and elevated structures. A somewhat similar work was proposed in [6], however there a 2D clustering scheme is deployed for detection that suffers when attempting to detect objects under elevated structures, unless highly restricted dimension filters are used. Moreover, the Joint Probabilistic Data Association scheme is adopted without explicit consideration of the combinatorial explosion phenomenon, a major bottleneck in cluttered environments. Another approach presented in [7], used JPDAF to track a limited number of dynamic objects detected by comparing consecutive LiDAR point clouds. Similarly, [8,9] exploit single LiDAR measurement patterns for object classification that bars the use of multiple LiDARs and require additional computational resources. The methods proposed in [8,10] are intended for stationary reference frames, whereas the work presented in [11] only considers tracking pedestrians.

The remainder of the paper is structured as follows: in Section 2, an overview of the MODT paradigm is presented. The target platforms for MODT implementation are introduced in Section 3, along with LiDAR sensor setup. The detection component of the proposed MODT scheme is laid down in Section 4. The Bayesian filter-based tracking module is described in Section 5, along with its mathematical derivation. The evaluation metrics for evaluation and benchmarking results are detailed in Section 6. The discussion on our evaluation results of the proposed framework in contrast with previous works is carried out in Section 7, followed by conclusions in Section 8.

## 2. MODT Overview

The objective of MODT is to determine the trajectories of detected objects and maintenance of their unique identity throughout the input sequence. The MODT methods are generally categorized into online and batch methods. Online methods rely on past frames up to the present frame information to solve tracking problems, whereas, batch methods utilize the entire sequence or the information from the future frames to assign the detections iteratively. Despite the fact batch methods are superior in accuracy, online methods are preferred for real-time applications, as tracking results are instantly available in the current frame [12]. Although recent advances in visual object detectors help provide promising tracking performances, they still tend to fail in application areas where limitations pertaining to cameras as primary sensors and low computational resource exist.

A feasible substitute to address camera-related limitations is to use LiDAR as a primary source. LiDAR provides a sparse spatial information of the environment that imposes less of a computational burden, making it viable for onboard computers. However, this solution comes with a tradeoff against the accuracy of object detection and classification. In addition, sparse and occluded information give rise to measurement uncertainty and a number of false detections. This requires additional treatment of detected objects for appearance-related attribute estimations. In MODT, data association of object detection in consecutive frames play an essential role in maintaining a correct trajectory and unique identity. To perform best associations, track motion models [13] and the appearances of detections are normally utilized [14].

**Figure 1:**
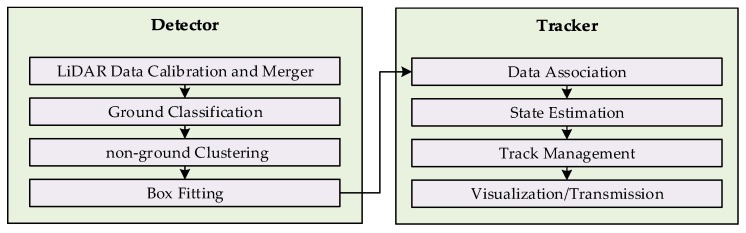
Multiple Object Detection and Tracking Architecture.

The uncertainties pertaining to the measurements and state estimations are generally handled by Bayesian probabilistic approaches (e.g, [12]). The unmodeled dynamics of the tracked object contribute in the uncertainties, generally tackled by Kalman filters [15] and particle filters [16], whereas, uncertainties due to measurements are treated by probabilistic data association methods, such as Joint Probabilistic Data Association filters [6] and Multiple Hypothesis Tracking [17]. The maintenance of the tracking information and pruning of the spurious and duplicate tracks is carried out by additional mechanism for track management. The proposed framework for LiDAR MODT is composed of two main components—detector and tracker—as shown in Figure 1. The detector component gets raw LiDAR data and produces a structured data that describes the attributes of the detected objects. The process of extracting trackable objects information in the detector module, supported by the related works is explained in Section 4. The tracking module based on the information provided by detector, performs tracking and enhances the object attributes with tracking information (track, location, speed, and motion type). The process followed by the tracking component in the proposed framework along with discussion on the related works is expressed in Section 5.

## 3. Platforms for MODT Implementation

**Figure 2:**
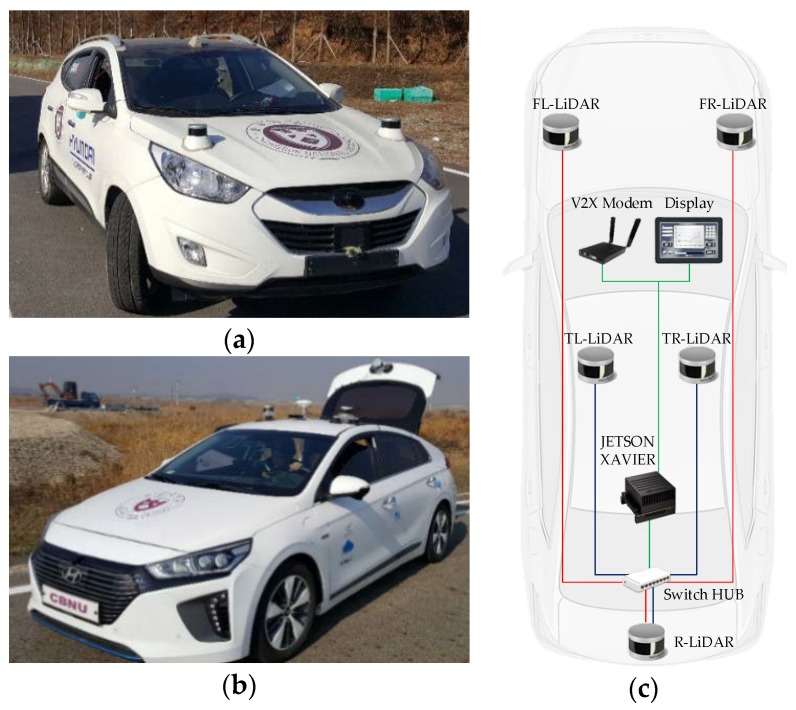
(**a**) TUCSON Platform; (**b**) IONIQ Platform; (**c**) LiDAR setup (connections in red-green are for the TUCSON and blue-green are of the IONIQ).

The proposed MODT architecture is implemented and tested on two platforms, a TUCSON and an IONIQ by Hyundai Motors (Hyundai Motor Company, Seoul, South Korea), shown in Figure 2a,b. Both platforms are equipped with three 16-channel Velodyne LiDARs (Velodyne LIDAR, San Jose, CA, USA), Figure 2c shows the connectivity and sensor locations. In the TUCSON, two LiDARs that measure the sides and front are mounted near the mid-fender region, whereas a single LiDAR is mounted on the top of trunk with a tilt to measure vehicle back that contributes in the height factor of elevated structures ahead of the vehicle. Similarly, two LiDARs are mounted on the top of the IONIQ with a tilt to measure the sides and front of the vehicle, and one is attached on the trunk to provide rear measurements. The LiDAR sensors provide measurements to a Jetson AGX Xavier unit by Nvidia (Nvidia Corporation, Santa Clara, CA, USA), via Ethernet. The embedded board processes the LiDAR information for MODT and provides tracking visualization to the display. Both platforms are equipped with V2X modems, that can be used to transmit the selected tracking information.

The computational environment used for developing and evaluating the proposed MODT framework is constituted by a desktop computer having an Intel Core i7-7700 CPU with 16 GB of RAM, running Robot Operating System (ROS) “Kinetic Kame” middleware on top of Ubuntu 16.04.5 LTS. Moreover, Jetson AGX Xavier is used for the computation time evaluations, that runs ROS “Melodic Morenia” middleware on top of Ubuntu Linux 18.04.1. Furthermore, parallel processing or GPU support on both computing environments is avoided.

## 4. Object Detection

The detection component of the proposed MODT scheme pre-processes the LiDAR point cloud into cloudlets that correspond to potential trackable objects. The pre-processing stage includes estimation of initial parameters and bounding box fitting. This information is fed forward to the tracker module for further processing. The detection component operates on a series of steps including, LiDAR calibration, ground extraction, point cloud clustering, and bounding box fitting, as shown in Figure 1.

### 4.1. LiDAR Calibration

The platforms used in this work are equipped with three VLP-16 Velodyne LiDAR sensors. Each sensor generates point cloud with respective reference frame. The transformation matrices are estimated by the procedure expressed in [18], such that, the point clouds are referenced to the center of platform. At each time step, the point clouds acquired from the LiDARs are transformed to the ego-vehicle center and are merged into a single point cloud. The LiDAR calibration step produces a single point cloud of calibrated LiDARs that is fed to the ground extraction module.

### 4.2. Ground Classification

The ground classification is referred to as the process of separating ground and non-ground point clouds. Methods to address this task are categorized as: (a) height threshold-based [19], (b) grid-based [20,21], (c) scan-based [22], and (d) learning-based [23]. Height threshold-based approaches operate on the assumption of a flat ground, as point clouds below a threshold level are considered as ground. Grid-based methods save computation time by dividing the point clouds into grid cells, enabling one to perform slope tests for specifying ground and non-ground grid cells/points. Scan-based methods assume a single LiDAR as a sensor and measure the distance from consecutive measurement rings. Learning-based methods utilize machine learning-based approaches to learn individual laser scan as an image to annotate the ground regions.

The task of ground classification from point clouds as a pre-processing for MODT systems has recently been approached by the abovementioned methods [19,20,21,24,25,26,27]. The scenario of fused point clouds is addressed in [28], that follows grid-based approach, however with plain ground assumption. Similarly, [21] incorporates a polar grid approach, but segments the ground with a threshold limit. Other approaches (e.g., [24,25,26]) consider a non-planar scenario in which each vertical slice consisting of all the points captured at the same moment by all laser rays, is analyzed separately. However, these methods cannot perform on the fused point clouds of multiple LiDAR sensors. Furthermore, the same limitation is faced in the methods [24,25], where scan segment matching of different channels of LiDAR data is used for ground segmentation. The short comings of above mentioned techniques are addressed by using inference in Markov Random Field (MRF) [29], and by a Convolutional Neural Network (CNN)-based approach [23]. The MRF-based approach shows robust results but at the cost of an enormous execution time of 145 s per frame of a Velodyne HDL-64E. On the other hand, CNN-based approaches segment the ground with GPU support in 7 ms per frame, but require tedious efforts for training the network.

**Figure 3:**
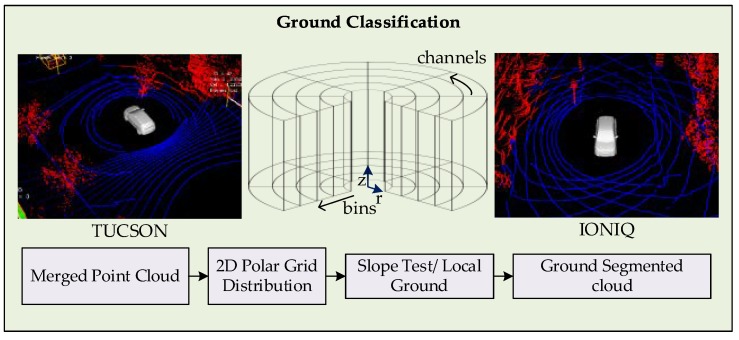
2D Polar Grid-based Point Cloud Distribution and Ground Classification.

The approach adopted in this work is a grid-based approach that considers a non-planar ground. The flow of the process followed in this module is shown in Figure 3. The merged point cloud obtained from the calibration module is treated to remove invalid and out of range points. The indexes of filtered cloud points are distributed into a cylindrical polar grid, where the polar grid is composed of channels, that are the vertical slices from the origin to the farthest possible readings. The channels are further segmented into bins, containing the possible number of points of that region. All the bins in the grid are traversed to estimate the local ground level, starting from the vehicle where ground level equals the sensor height. The ground levels in the adjacent cells are estimated based on lowest point, height of bin, and absolute slope to the adjacent bin. The local ground level divides the points into ground and non-ground points at bin level. Fine correction is performed to remove the points pertaining to the bins with avoidable threshold height, such as vegetation. The indexes of points referring to non-ground points are used to filter the cloud and is fed forward for further processing.

A similar implementation for ground classification is adopted in [6], however with an additional step of consistency check along the channels. This step is not necessary in this implementation because every bin gets relatively precise local ground information. Furthermore, the key factor that enhances the performance is to use points’ indexes in grid distribution, avoiding the whole cloud conversions between polar and rectangular coordinates.

### 4.3. Point Cloud Clustering

The point cloud clustering step is the process of grouping and labeling the points possibly pertaining to a single object [30]. The concept has a diverse application area and so are the methods. In general, clustering algorithms may be divided into: (a) hierarchy-based [6], (b) centroid-based [30], (c) distribution-based [31], and (d) density-based methods [32]. The hierarchy-based or connectivity-based algorithms operate on the distances from the neighboring measurements. The hierarchical nature allows the expansion and number of clusters based on distance function or linkage criteria used. Furthermore, hierarchical methods can be agglomerative or divisive, by having single or multiple starting positions for clustering. Moreover, these approaches exist under 3D [22,33], 2D [34,35] and 2.5D [4] configurations. Where clustering region grows in 3D, 2D, or in 2D with height information for 2.5D clustering. However, these methods are not robust towards outliers and may also result in additional partitions or merger of clusters. Therefore, care needs to be taken in choosing appropriate clusters. In this work, the presence of appropriate filters is assumed to handle the outliers.

The centroid-based algorithms [30] include K-means, Gaussian Mixture Models, Fuzzy c-mean, and similar expectation maximization variants that require prior knowledge of the number of clusters. In addition, the performance of these algorithms relies on process initialization. These traits of centroid-based algorithms bar their use in this work, as the number and position of objects are not known. The distribution-based methods [31] are related to the use of distribution models in statistic, like Gaussian and Normal. These methods can provide information beyond the cluster assignments of objects but at the cost of overfitting issues, especially if complexity of model is not constrained. Moreover, such methods tend to fail in the absence of models in the database for optimization. Hence, application of this technique would be cumbersome in this work. The density-based methods [32] operate by identifying the high-density regions to cluster. These approaches deploy a local cluster criterion, resulting in arbitrary shape and point distribution of clusters. The LiDAR measurements become sparse with the increase in distance from the sensor. This inherent property of LiDAR limits the use of density-based methods for clustering.

**Figure 4:**
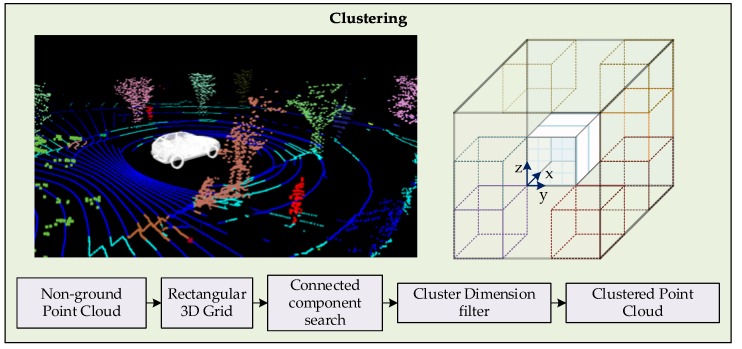
3D rectangular grid-based clustering of point cloud, trackable objects in red color.

In this work, the clustering of point clouds is carried out through a hierarchy-based method. The flow of the clustering process is shown in Figure 4. Firstly, the point cloud without ground points is distributed into a rectangular 3D grid. The consideration of a 3D grid is made to cluster the objects that exist under elevated structures, such as road lights, traffic signals, bridges and tree branches. In urban scenarios with a tilted multiple LiDAR setup, 2D grid approaches are inefficient for clustering. Later the grid is treated with a 3D connected component clustering, formulated from a binary image clustering method.

An index cell is selected, and its 26 adjacent cells are searched for a minimum number of points. The qualifying neighboring cells are marked as the cluster members and are indexed for subsequent search. Labeling of cells are carried out to distinguish the clusters and to ensure that every cell is searched only once. The time complexity of clustering module is handled by setting a threshold limits x, y, and z dimensions. The x dimension is the heading of vehicle in the LiDAR sensor frame, the positive and negative limits are set based on the maximum distance at which identifiable cluster can be formed. Similarly, y dimension limits are set to a distance that minimizes the clustering of outliers like buildings and vegetation, considering a reasonable distance for crossroads. Moreover, limits in the z dimension are set to be little higher than the tallest trackable object. With these clustering limits in place, clustered point cloud is efficiently obtained.

The point cloud segmented into clusters in a 3D grid is converted into clustered point clouds, where each cluster is associated to a potential trackable object. The dimension filter is used to filter out the outliers and to classify the objects. Furthermore, elevation and number of points of a cluster are also considered as parameters. First, clusters with dimensions exceeding the smallest and largest trackable objects’ dimensions are filtered out. Similarly, elevated clusters not satisfying the ground threshold limits are filtered. The clusters qualifying for trackable objects are proceeded to the bounding box fitting and classification stage.

### 4.4. Box Fitting and Object Classification

The box fitting and classification of objects in a LiDAR point cloud is a challenging task, mainly because of occlusions, that is any obstruction in the line of sight of LiDAR. Box fitting of point clouds not only helps in finding the objects’ centroids that can be efficiently tracked but also provides an initial pose estimation. This task is addressed by either (a) model-based, or (b) feature-based approaches. Model-based estimations tend to match the raw point cloud with a known geometric model [36]. The pose estimation using model-based methods offer optimal results, however, at a cost of high computational burdens. On the other hand, feature-based methods rely on edge features to deduce the pose of an object [9,11,19,37]. The feature-based estimations are computationally efficient; however, these are sensitive to unstable measurements. Other works have also considered learning based neural network models to estimate the object pose [38], by training the detectors from all possible view angles. In this work, minimum rectangle area [39] with L-shape cloud fitting [9] is utilized as in [13,40], with optimized computational and accuracy considerations.

**Figure 5:**
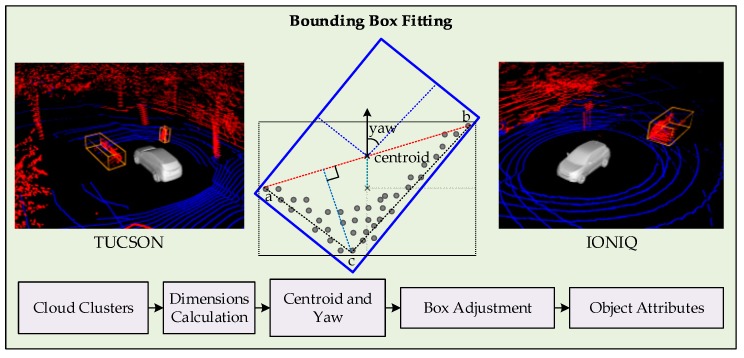
Dimensions, centroid, and yaw estimation of cluster for bounding box fitting and measurement generation.

Since the objects in LiDAR point cloud measurements are always under occlusion, the minimum rectangle area alone cannot serve the purpose of bounding box fitting, and eventually affect the incorrect pose estimation. Hence, L-shape fitting is carried out over minimum area rectangle fitting to correct the pose of box [9], as shown in Figure 5.

First, the dimensions of the clustered point cloud are calculated where length and width specify the initial area of the fitting rectangle in 2D. Then, the farthest two points of the clusters are found and a line between them is calculated. Finally, the most deviated point from this line is examined within the clustered cloud. This results in three points that correspond to the three corners of the actual pose of object. The rotation of the rectangle or pose of the object is estimated by calculating the angle between the initial edge of the rectangle and to the line joining the farthest point from the diagonal line to its either end. The height information is used as calculated from the dimensions of the point cloud to form a 3D bounding box. This procedure results in a box that represents an object in the LiDAR point cloud, and the location of object is indexed to the centroid of the box. At the end, the dimensions of the box are used to classify the object as vehicle or pedestrians for tracking initialization. For visualization, the bounding boxes matching strict dimension rules are replaced with 3D CAD models. In summary, considering the possibility of multiple LiDARs being used for measurements, the extracted clouds are calibrated and merged, such that all the measurements are referenced to the center of the ego-vehicle. Slope-based approach is adopted to segment the point cloud into ground and non-ground measurements. The point cloud pertaining to the non-ground point cloud is treated with a 3D connected component method for clustering. Subsequently, the filtered clusters of the point cloud that are potential trackable objects, are processed for box-fitting. A combination of minimum area rectangle and L-shape fitting process is applied to fit a 3D bounding box with an estimated pose of an object. The last step of the detection component is the assignment of classes based on the strict dimension criteria. The result of the detection component is a list of bounding boxes of known position, centroids, pose, and possibly class of the objects, that are fed as measurements to the tracking component for further processing.

## 5. Tracking

The tracking component of the proposed framework uses the measurements from the detection component to determine the location, path, heading angle, velocity, and angular velocity of the detected objects. Moreover, all the tracked objects essentially need to use a unique identity. As in the context of environmental perception, the said measurements are useful only if these can be incorporated in the ego-vehicle’s control and decision making. The task of tracking objects in the urban scenario using LiDAR need to address several challenges including, occlusion, dissimilar motion patterns of objects, and clutter. The term occlusion refers to the possibilities of incomplete spatial information either due to the pose of the ego-vehicle, or an object obstructing the measurement. This may result in partial or complete occlusion of an object. The partial occlusion can be handled by the detection component of the framework. Also, a complete obstruction of the tracked object contributes to the location uncertainty. In addition, the objects in the urban scenario tend to follow dissimilar motion patterns, like consistent variation in pose and speed. Moreover, many objects can exist close to each other forming clutter, contributing in the rise of uncertainty in the measurements.

**Figure 6:**
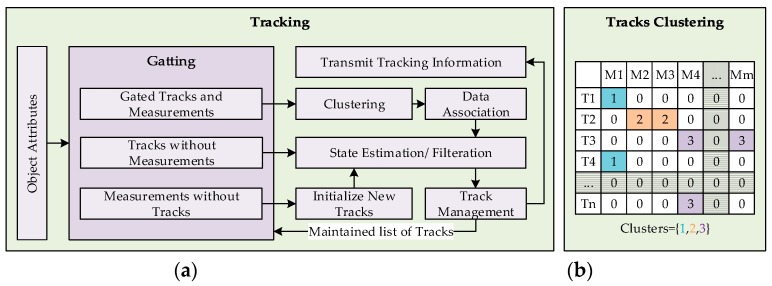
(**a**) Tracking module architecture; (**b**) Clustering example of joint associated events with tracks T and measurements M.

The tracking component needs to address the presence of two prominent uncertainties that are due to motion and clutter. Hence, two filters are set in place to address the both uncertainties while tracking. The filter for motion uncertainties incorporates a system with multiple motion models to track objects with uncertain motion patterns. Whereas, the clutter filter utilizes probabilistic data association approach to address uncertainties that arise when the tracked objects are closely located. Both mentioned filters however can be combined into a single tracking scheme with filtration performed at different steps. A similar tracking approach is adopted in [2,13,34,40]. In the proposed framework, Interacting Motion Model (IMM) is deployed to lever dissimilar motion patterns of objects that is coupled with Unscented Kalman Filter (UKF) to handle non-linearities of models. Whereas, the Joint Probabilistic Data Association Filter (JPDAF) is utilized to address the uncertainties due to clutter, applied at the data association stage of the UKF. The filter encompassing the individualities of both the filters is referred to as IMM-UKF-JPDAF. The similar arrangements of IMM-UKF-PDA [34], IMM-UKF-MHT [17], and IMM-PF [16], also exist in the literature. The tracking component shown in Figure 1 is further elaborated in Figure 6a that describes the flow of information in the proposed architecture. The working process of the tracking component is detailed in the subsequent subsections.

### 5.1. IMM-UKF-JPDAF

The implementation of IMM-UKF-JPDAF is an approach to efficiently address the problem of recursively estimating states and mode probabilities of targets, described by a jump Markov non-linear system, in the presence of clutter. Let the non-linear stochastic state space model be represented by Equations (1) and (2), forming a set of r models $M={\left\{M_{j}\right\}}_{j=1}^{r}$:

(1) $$x_{k+1}=f_{j}\left(x_{k},u_{k}\right)+w_{j,k},$$

(2) $$z_{k}=h_{j}\left(x_{k},u_{k}\right)+v_{j,k}.$$

where, the input vector is $u_{k}\in {\mathbb{R}}^{p}$, measurement vector is $z_{k}$, whereas f and h represent system and measurement functions, respectively. Moreover, w and v characterize zero-mean Gaussian noise sequences, which are mutually independent covariance matrices Q and R, respectively. The progression of the system among r models is considered as first order Markov chain that operates on the top of time-invariant Markovian model transition probability matrix:

(3) $$\Pi =\left(\begin{array}{ccc} p_{11} & \cdots & p_{r1}\\ \vdots & \ddots & \vdots \\ p_{1r} & \cdots & p_{rr} \end{array}\right)\in {\mathbb{R}}^{r\times r}.$$

The elements $p_{ij}$ of matrix in Equation (3) represent mode transition probability from model i to j as expressed in [41]. A similar approach of IMM-UKF-PDA is explained in [34] as a five-step process. In this work, the difference is at the data association stage, as multiple targets are to be tracked instead. The five steps of the process are: (a) interaction, (b) state prediction and measurement validation, (c) data association and model-specific filtering, (d) mode probability update, and (e) combination step. The process of each step is briefly explained along with mathematical representations.

#### 5.1.1. Interaction Step

In this step, the initial state and covariance corresponding to each model filter is probabilistically mixed with the state and covariance of the previous time stamp:

(4) $${\hat{x}}_{j,k-1}^{⋆}=\overset{r}{\underset{i=1}{\sum }}\mu_{\left(i|j\right),k-1}{\hat{x}}_{i,k-1},$$

(5) $${\hat{P}}_{j,k-1}^{⋆}=\overset{r}{\underset{i=1}{\sum }}\mu_{\left(i|j\right),k-1}\left[P_{i,k-1}+\left({\hat{x}}_{j,k-1}-{\hat{x}}_{j,k-1}^{⋆}\right){\left({\hat{x}}_{j,k-1}-{\hat{x}}_{j,k-1}^{⋆}\right)}^{T}\right].$$

In Equations (4) and (5), $\mu_{\left(i|j\right),k-1}$ represent conditional mode probabilities, that imply that the system has transited from mode i in the previous time stamp to mode j in the current time cycle, and is calculated by:

(6) $$\mu_{\left(i|j\right),k-1}=\frac{P_{ij}\mu_{i,k-1}}{\sum_{i=1}^{r}{\bar{\mu }}_{i,k-1}}.$$

The conditional mode probabilities depend on the priori mode probabilities, ${\bar{\mu }}_{k}={\left({\bar{\mu }}_{1,k},\ldots ,{\bar{\mu }}_{r,k}\right)}^{T}$ of the current frame, which is described by the prediction of mode probabilities of previous frame and elements of matrix Π in Equation (3).

#### 5.1.2. State Prediction and Measurement Validation Step

Once the initial states and covariances are obtained from the interaction step, prediction step is executed. Each model individually predicts the current state through respective stochastic non-linear model. Due to non-linearities, UKF is deployed to estimate the predicted states and covariances [15]:

$$X_{k-1}=\left(\begin{array}{ccc} {\hat{x}}_{k-1}^{⋆} & \gamma \sqrt{P_{k-1}^{⋆}}⊕{\hat{x}}_{k-1}^{⋆} & -\gamma \sqrt{P_{k-1}^{⋆}}⊕{\hat{x}}_{k-1}^{⋆} \end{array}\right),$$

$$X_{i,k}^{⋆}=f\left(X_{i,k-1},u_{k-1}\right),\;i=0,\ldots ,2n$$

(7) $${\bar{\hat{x}}}_{k}=\overset{2n}{\underset{i=0}{\sum }}w_{i}^{m}X_{i,k}^{⋆}$$

(8) $${\bar{P}}_{k}=\overset{2n}{\underset{i=0}{\sum }}w_{i}^{c}\left(X_{i,k}^{⋆}-{\bar{\hat{x}}}_{k}\right){\left(X_{i,k}^{⋆}-{\bar{\hat{x}}}_{k}\right)}^{T}+Q_{k-1}.$$

$$X_{k}=\left(\begin{array}{ccc} {\bar{\hat{x}}}_{k} & \gamma \sqrt{{\bar{P}}_{k}}⊕{\bar{\hat{x}}}_{k} & -\gamma \sqrt{{\bar{P}}_{k}}⊕{\bar{\hat{x}}}_{k} \end{array}\right),$$

$$K_{i,k}=h\left(X_{i,k},u_{k}\right),\;i=0,\ldots ,2n$$

(9) $${\bar{\hat{z}}}_{k}=\overset{2n}{\underset{i=0}{\sum }}w_{i}^{m}K_{i,k}$$

(10) $$S_{k}=\overset{2n}{\underset{i=0}{\sum }}w_{i}^{c}\left(K_{i,k}-{\bar{\hat{z}}}_{k}\right){\left(K_{i,k}-{\bar{\hat{z}}}_{k}\right)}^{T}+R_{k}.$$

Initially, 2n+1 sigma points $X_{i}$, with i=0,…2n in the matrix form $X=\left(X_{0},\ldots ,X_{2n}\right)\in {\mathbb{R}}^{n\times \left(2n+1\right)}$ are chosen by square root decomposition of mixed initial covariance of each filter. Each sigma point possesses two scalar weights $w_{i}^{m}$ and $w_{i}^{c}$ given by:

(11) $$w_{0}^{m}=\frac{\lambda }{n+1},\;w_{0}^{c}=\frac{\lambda }{n+1}+\left(1-\alpha_{U}^{2}+\beta_{U}\right)$$

(12) $$w_{i}^{m}=\;w_{i}^{c}=\frac{\lambda }{2\left(n+\lambda \right)},\;i=1,\ldots ,2n$$

(13) $$\lambda =\alpha_{U}^{2}\left(n+\kappa_{U}\right)-n$$

(14) $$\gamma =\sqrt{n+\lambda }$$

The terms $\alpha_{U}$, $\beta_{U}$, $\kappa_{U}$, λ, and γ in Equations (11)–(14) are scalar parameters. These sigma points are treated with the system function f, whereas mean and covariance is retrieved through calculated weights. Subsequently, the predicted mean and covariance together with the $Q_{k-1}$ are used to find new sigma points. These points are propagated through the function h. This provides the priori measurement ${\bar{\hat{z}}}_{k}$ along with the associated innovation covariance matrix $S_{k}$ from the propagated sigma points $K_{i}$ for each model.

Now, consider tracks $T_{p,k}={\left\{q_{j,k}\right\}}_{j=1}^{T}$ of objects at time step k, with N measurements set $Z_{l,k}={\left\{z_{i,k}\right\}}_{i=1}^{N}$. A measurement is considered valid if it lies inside the elliptical validation gate G(q) of one target q. The validation gate of an object q is taken to be same for all models in M and the model resulting in largest area is chosen. The validation gate for each track q is given by:

(15) $$G\left(q\right)=\left\{y_{k}=z_{k}:{\left[z_{k}-{\bar{\hat{z}}}_{i_{q},k}\right]}^{T}S_{i_{q},k}^{-1}\left[z_{k}-{\bar{\hat{z}}}_{i_{q},k}\right]\leq \gamma_{G}\right\}\;i_{q}≔argmax_{i\in M}\det\left(S_{k}^{i}\right)$$

The gate is entered at ${\bar{\hat{z}}}_{i_{q},k}$, where $i_{q}$ is the index of the model in the model set that corresponds to the largest residual covariance. Moreover, the gate threshold $\gamma_{G}$ is obtained from the inverse chi-square cumulative distribution with a gate probability $P_{G}=P\left(z_{k}\in G\left(q\right)\right)$. Consequently, a set of validated measurements is obtained $Y_{v,k}={\left\{Y_{i,k}\right\}}_{i=1}^{N_{k}}$ for all models of an object q, such that $N_{k}\leq N.$

#### 5.1.3. Data Association and Model Specific Filtering Step

The key operation of JPDAF is the definition of marginal events $\theta_{j,q}$ and evaluation of corresponding conditional joint probabilities. A marginal association event is said to be effective when a measurement $y_{j,k}$ is associated with an object q, such that $y_{j,k}\in G_{k}\left(q\right)$. Whereas a joint association event $\Theta =\cap_{j=1}^{N_{k}}\theta_{j,q_{j}}$ occurs when multiple measurements $y_{j,k}$ get validated for a track $q_{j}$. Based on this information, a validation matrix $\Omega =\left[\omega_{jq}\right]$ can be computed. Here, $\omega_{jq}$ is a binary variable describing that the measurement j lies in the validation gate of track q in an association event θ. The validation matrix can also be used to represent joint association event in a matrix form $\hat{\Omega }=\left[{\hat{\omega }}_{jq}\right]$, such that:

(16) $${\hat{\omega }}_{jq}=\{\begin{array}{cc} 1 & if\;\theta_{j,q}\subset \Theta \;\\ 0 & otherwise \end{array}.$$

Once the association matrix is populated, hypotheses of all possible occurrences of events are generated, including the consideration of measurement being a false alarm. In the event of a large association matrix, where the association hypothesis grows in number, this causes excessive computational burden, known as the combinatorial explosion problem. To mitigate this issue, a clustering technique is deployed in this work to limit the hypothesis building to a cluster of associated events. Figure 6b shows an association matrix with clustered associations. The number of clusters is equal to the sum of marginal and joint association events. Clusters pertaining to the marginal events contain single measurements to track association, the hypothesis of which involves false positive probability, whereas, hypotheses for clusters of joint association events compute probabilities of all possible combinations of association probabilities, including the possibility of measurement being a false positive. The clustering technique helps in reducing the number of hypothesis combinations that naturally grows in cluttered environments. Furthermore, the spread of covariance of a track prediction increases if no measurements are associated in consecutive time frames, also increasing the gate area for association. The larger gate area also results in larger number of joint association events.

Upon generation of hypothesis for all possible occurrences of events within the respective clusters, marginal association probabilities are computed that is the probability sum of the joint association events given that the measurement j belongs to track q:

(17) $$\beta_{jq}^{cl}=\underset{\Theta^{cl}}{\sum }P\left\{\Theta^{cl}|z_{k}\right\}{\hat{\omega }}_{jq}^{cl}\left[\Theta^{cl}\right],\;j=1,\ldots ,N^{cl}\;and\;q=1,\ldots ,T^{cl},$$

(18) $$P\left\{\Theta^{cl}|z_{k}\right\}=\frac{1}{c}\overset{N^{cl}}{\underset{j=1}{\prod }}g_{jq}P_{D}\overset{T^{cl}}{\underset{q=1}{\prod }}{\left(1-P_{D}\right)}^{\delta_{q}}\;\overset{N^{cl}}{\underset{j=1}{\prod }}\beta^{\phi_{j}}.$$

Here, $g_{jq}$ is the likelihood of measurement j given the track q. Whereas, c is the normalizer, while $\delta_{q}$ and $\phi_{j}$ represent the number of unassigned tracks and measurements, respectively, within the cluster cl. Subsequently, weighted measurement residual ${\tilde{z}}_{i,q,k}$ is computed for each corresponding model i of filter according to the associated measurement set:

(19) $${\tilde{z}}_{i,q,k}=\overset{N^{cl}}{\underset{j=1}{\sum }}\beta_{j,q}^{cl}{\tilde{z}}_{i,j,q,k}$$

To calculate the Kalman gain $K_{k}$, cross covariance matrix $C_{x_{k},z_{k}}$ is evaluated together with the innovation matrix $S_{k}$ for each model of the corresponding track to update state and covariance matrices of tracks:

(20) $$C_{x_{k},z_{k}}=\overset{2n}{\underset{i=0}{\sum }}w_{i}^{c}\left(X_{i,k}-{\bar{\hat{x}}}_{k}\right){\left(X_{i,k}-{\bar{\hat{z}}}_{k}\right)}^{T}\;K_{k}=C_{x_{k},z_{k}}S_{k}^{-1}$$

(21) $$x_{i,q,\left(k|k\right)}=x_{i,q,\left(k|k-1\right)}+K_{i,q,k}{\tilde{z}}_{i,q,k}$$

(22) $$\begin{array}{c} P_{i,q,\left(k|k\right)}=P_{i,q,\left(k|k-1\right)}-\left(\overset{N^{cl}}{\underset{j=1}{\sum }}\beta_{j,q}^{cl}\right)K_{i,q,k}S_{i,q,k}K_{i,q,k}^{T}\\ \;+K_{i,q,k}\left[\overset{N^{cl}}{\underset{j=1}{\sum }}\beta_{j,q}^{cl}{\tilde{z}}_{i,j,q,k}{\tilde{z}}_{i,j,q,k}^{T}-{\tilde{z}}_{i,q,k}{\tilde{z}}_{i,q,k}^{T}\right]K_{i,q,k}^{T} \end{array}$$

#### 5.1.4. Mode Probability Update Step

This step updates the mode probabilities $\mu_{q,k}$ based on the best fitting measurements [41], using Gaussian-uniform mixture model likelihoods $\lambda_{j,k}$, computed as $N\left({\tilde{z}}_{q,k};0,S_{q,k}\right)$:

(23) $$\mu_{q,k}=\frac{{\bar{\mu }}_{q,k}\lambda_{j,k}}{\sum_{i=1}^{T}{\bar{\mu }}_{i,k}\lambda_{i,k}},\;where\;\lambda_{j,k}\;\begin{array}{c} asm\\ \tilde{=} \end{array}\;N\left({\tilde{z}}_{q,k};0,S_{q,k}\right)$$

#### 5.1.5. Combination Step

IMM-UKF-JPDAF recursively estimate the states, covariances, and mode probabilities with the help of individual model likelihoods. In the last step of the tracker, the individual filter states and covariances are combined to a single weighted output using mode probabilities of tracks:

(24) $${\hat{x}}_{q,k}=\underset{i}{\sum }x_{i,q,k}\mu_{i,q,k}\;P_{q,k}=\underset{i}{\sum }\left[P_{i,q,k}+\left({\hat{x}}_{q,k}-x_{i,q,k}\right){\left({\hat{x}}_{q,k}-x_{i,q,k}\right)}^{T}\right]\mu_{i,q,k}$$

### 5.2. Track Management

The tracking paradigm requires an efficient management mechanism to cater to the associated uncertainties and to maintain and provide actionable information. The main purpose is to maintain the tracks of objects that undergo a complete occlusion and to perform track fragmentation on reappearance. Furthermore, pruning of tracks pertaining to false positive measurements is desired. The tasks associated with the module are briefly expressed in subsequent subsections.

#### 5.2.1. Track Initialization and Validation

Each track holds two numeric flag variables that represent the status of track, labeled as maturity and lost. The maturity flag counts the successful association of measurements in consecutive time steps, while the lost flag counts the consecutive timesteps where no measurement gets associated with a track. When the track gets initialized, both the flags are set to zero and later incremented accordingly. Track is validated if the maturity count gets greater than five. This implies that a track must get measurements in at least five initial consecutive time steps, to be considered as valid. The matured tracks that stop getting any measurements associated for 20 consecutive time steps are lost and their tracking is discontinued. In this duration the object is assumed to temporarily occluded. The prediction of track in lost status is made with an assumption that the occluded object will retain its mode, heading, and speed. As the track enters in lost status, the uncertainty starts to increase and retaining a track beyond 20 frames have higher chances of producing incorrect associations.

#### 5.2.2. Track Pruning

A check is set in place to ensure that multiple tracks do not get associated with the same object (duplicate tracks) for more than five consecutive time steps. The states of all tracks are traversed in every time step with threshold Euclidian distance limit of less than one meter. Among the qualifying set of tracks only the track with highest maturity count is retained and rest are pruned out.

#### 5.2.3. Kinematic Parameters Update

The state space models cannot accurately update the parameters of yaw, velocity, and angular velocity. For this matter, the track history is maintained, and previous five states’ information is utilized to estimate them. Moreover, the first five frames after initialization of the track ensures more exact parameters estimations of newly validated track. The heading information from the detector alone cannot be relied due to the phenomenon of occlusion. Hence, the heading of an object is estimated from both, the detector and the track history of the object, making it more reliable. Therefore, at every update step, the kinematic parameters estimated by incorporating the track history are integrated into the object states for more accurate prediction in the subsequent time step.

In summary, an IMM-UKF-JPDAF is implemented as a combination of two filters to address the uncertainties pertaining to occlusions and clutter. The JPDAF shortcoming of combinatorial explosion is approached by mitigation strategy of clustering. The tracking component gets the object attributes as a structured data and through recursive operation of the proposed architecture estimates updated object states and tracks, covariances, and modes with respect to ego-vehicle reference frame. The object states are represented by position of object in a 2D space, heading of object, and linear and angular velocities. Whereas covariances and modes describe the degree of uncertainty and motion pattern (static, linear or angular) respectively. This data is managed and maintained through a track management module to produce an actionable information in a computationally efficient manner and can be shared across the platform over V2X.

## 6. Evaluation

The method for evaluating this framework is of MOT16 as proposed by [42]. However, metrics pertaining to the classification of objects are excluded, as they are more associated to visual tracking schemes. The metrics that are used for evaluations are: (a) tracker to target assignment, (b) multi object tracking accuracy A, (c) multi object tracking precision P, and (d) track quality. The metric for tracker to target assignment defines the reliability of tracking algorithm by considering the number of False Positives FP, False Negatives FN, and ID Switches IDSW. Where, FP and FN deals with incorrect associations of the measurements. Furthermore, the  IDSW determines the number of ID switches across all the fames for an object, also termed as fragmentation of a tracked object. The metric for A is computed by:

(25) $$A=1-\frac{\sum_{t}\left(FN_{t}+FP_{t}+IDSW\right)}{\sum_{t}G_{t}},$$

where, t is the frame index and G is ground truth value. The negative A indicates that the number of errors has exceeded the actual number of objects, maximized at 100. Furthermore, the metric P is evaluated by the average Euclidean distance between the estimated track instances and the ground truth. The metric for track quality is described by classification of a track into: Mostly Tracked MT, Partially Tracked PT, and Mostly Lost ML. This is carried out by comparing the extent of ground truth G trajectory recovered by the tracking algorithm. A target is MT if it is successfully tracked for at least 80% of its life span. Where, IDSW number is irrelevant in this metric, as the ID needs not to remain the same throughout the track. If the recovered track is for less than 20% of its total length, it is said to be ML. Other tracks fall under the class of PT. A higher number of MT and few ML is desirable.

To evaluate the tracking framework against non-synthetic data, KITTI datasets [43] are used. The datasets include camera image sequences along with synchronized Velodyne LiDAR point cloud data. For validation purposes, object labels are also provided that can be used as ground truth. However, the benchmark is intended primarily for visual tracking, hence objects are annotated only if they are visible in the camera frame. This limits the ground truth information for LiDAR to only the front of ego-vehicle. Furthermore, the Velodyne HDL-64E sensor used in KITTI datasets has the effective measurement range of 20–40 m [44]. Beyond this range, the measurements get sparse and object shapes become less recognizable. Since this work relies on LiDAR data alone for tracking, a comparable annotated ground truth information would be required for validation.

In this work, a criterion is proposed that overlaps the ground truth information pertaining to the camera and LiDAR frame as a reference. Hence a subset of ground truth is attained such that: (a) object is visible to both sensors (front of ego-vehicle), (b) existence of object within 30 m range from the sensor, and (c) the life time of a track is specified by the duration of first two conditions being true. The raw data provided by the KITTI datasets under the category of ‘City’, with the ground truth annotations are used for being more relevant to this implementation. However, the objects of type ‘Tram’, ‘Misc’, ‘truck’, and ‘person sitting’ are excluded for evaluation, and contribute to FP if detected and tracked.

### 6.1. Benchmarking Results

The KITTI Dataset provides ground truth for tracking information structured in XML format. Moreover, provides support to generate similar XML file directly from tracking algorithm. To perform the evaluation and benchmarking, the tracking component is programed to produce an XML file with tracking information similar in format to that of ground truth. In addition, MATLAB wrapper is also offered by KITTI Dataset to extract tracking information from the XML files to perform the metrics evaluations. The evaluation results of 10 dataset sequences are tabulated in Table 1, along with dataset recording number, frame count and the number of trackable objects that qualify the criterion defined in the previous subsection.

The evaluation metrics in Table 1 reflect that the MODT algorithm is reasonably accurate with an average A amounting to 87.8%. This is lowered mainly because of FP and FN, as IDSW rarely occur. The P metric shows that the tracker remains under a meter radius of the object on the average. Partial occlusion and multiple track measurements in the vicinity are the major contributing factors for the deviation. Similarly, the quality metrics on average establishes that on average 80% of the tracks fall under the category of MT, while PT and ML categories share under 10% of the weightage on average. The overall tracking quality is decreased mainly because of variations in the datasets, the lowest quality is resulted in the dataset 5, 9, and 51. These data sets also yield higher number of FP and FN that contribute in loss of track and higher deviation in P. Furthermore, the dataset 5 and 9 are common in existence of closely parked vehicles (filtered out by dimension filter). Moreover, the quality of algorithm suffers due to sudden changes in speed and sharp turns, resulting in the total of 5 IDSW.

### 6.2. Experiments on Platforms

**Figure 7:**
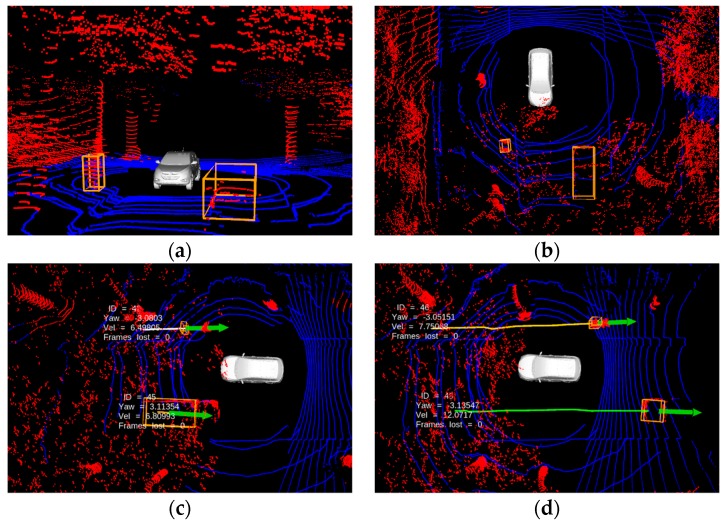
Detection and tracking of vehicles and pedestrians under the cover of tree branches: (**a**) front view; (**b**) top view; (**c**) tracking at initial stage; (**d**) mature tracks.

The datasets provided for evaluation purposes are generally based on single LiDAR sensor measurements, with limited vertical FOV. This restricts the intervention of elevated structures like traffic lights, bridges, and trees to be a part of LiDAR measurements within the effective horizontal range (20–40 m). However, measurements pertaining to these structures become significant when the sensor is tilted, or a combination of multiple sensors are used to capture most part of the surroundings. Furthermore, an assumption of height threshold for measurements contribute the similar problem to that of assuming a planer ground. That is, setting a vertical threshold limit for the height of measurement might work for a planer ground but suffer in a non-planer ground scenario. Here, an efficient clustering technique is required to detect and track an object that exists under the elevated structures. Addressing this issue is one on the main contributions of this work, as former works generally rely on vertical threshold limit. The proposed MODT method is tested on the platforms in the scenario where roadway is covered with trees on the top, while the trackable objects exist underneath. The limits for ground classification and clustering are set to 30 m from platform (horizontal x-y axis) and 10 m (vertical z axis). Figure 7a,b, show the detection of a pedestrian and a vehicle under the tree branches, whereas Figure 7c,d, show tracking path.

### 6.3. Execution Times

The tracking evaluations for datasets are carried out on desktop computer; however, time complexity is also measured on the Jetson board. The time consumed by individual components of the algorithm while executing in respective computational environment is presented in Table 2.

The overall performance of the algorithm in terms of metrics pertaining to accuracy, precision, and quality are comparable to state-of-the-art MODT paradigms. However, the datasets provided by KITTI Datasets are underprovided with elevated structures and non-planar grounds to test the true potential of the algorithm. On the other hand, the factors affecting the performance are the result of compromise made against the time complexity. Despite the optimized code, the execution times in Jetson exceeds the sampling time of LiDAR in datasets with increased number of tracked objects. This however is addressed by the tracker module, as the rate of prediction is coupled with the delay in arriving measurements.

## 7. Discussion

LiDAR-based MODT approaches are relatively scarce in literature compared to camera- based ones. Furthermore, LiDAR-based schemes are rarely evaluated on MOT16 metrics, except for the work reported in [6]. Therefore, a similar evaluation criteria and datasets are utilized to perform a fair comparison. However, instead of computing bounding box overlap for precision metric, Euclidean distance error is utilized, like the work of [8,9,10]. Because the objects detected through LiDAR measurements lack in prior dimensional information and provided ground truth does not specify frame-wise box dimensions. The evaluation metrics of these works are tabulated in Table 3.

The proposed architecture possesses competitive performance metrics to the relevant former work presented in [6]. The evaluations based on similar datasets yield better metrics of A, FN, and IDSW. The huge difference in IDSW percentage is because of gate size is adjusted to consider more measurements for data association. An additional track clustering mechanism is proposed to handle the combinatorial explosion problem for efficient data association. The higher FP percentage is an indicator of having more flexible dimension filter in the detection module. This is intentional to ensure that all potentially trackable objects under occlusion can be considered as measurements for the tracker. The average computation time in desktop environment is amounting to nearly a half. However, implementation on embedded computational environment of Jetson CPUs consumes similar computation times.

The framework proposed in [9] is evaluated on Wuhan Road and Wuhan University datasets recorded by a single 32-channel LiDAR sensor. The performance metrics show slightly lower percentages of A and FP, while remarkably better numbers in FN, IDSW and P. The precision metric is relatively higher than the proposed framework, mainly because of clustering scheme that fits geometric models to estimate the centroid. However, this method exploits the sensor rings from a single sensor and cannot guarantee similar performance in a multiple LiDAR scenario. Furthermore, the computation time on a desktop environment with half the density of point cloud consumes more than two folds to the proposed framework.

The work of [8] utilizes a generative object detection scheme, that again assumes a single LiDAR sensor. However, the proposed framework in comparison shows slightly better performance metrics, whereas, the work of [10] is focused on tracking pedestrians only, resulting in expected better precision metric. As centroid of pedestrian like object is not much affected even if partially occluded. Furthermore, both approaches are evaluated in an environmental setup with stationary sensor frame, where performance metrics are less affected by uncertainties due to sensor motion.

## 8. Conclusions

In this work, a computationally efficient MODT algorithm for a multi-LiDAR setup is proposed, which can perform in real-time on a vehicle embedded computer. The slope-based ground classifier considers a non-planar ground assumption. Furthermore, the object detector relies on a flexible dimension filter to classify trackable objects, where the LiDAR point cloud is efficiently clustered using a 3D connected component method, with an assumption of elevated structure presence. Moreover, the proposed combinatorial explosion-aware IMM-UFK-JPDAF-based tracker operates in the presence of uncertainties to perform efficient tracking. In addition, the MOT16 metrics established for performance evaluation are used to demonstrate state-of-the-art results against non-synthetic datasets. The main objective of this work was achieved with an implementation of MODT algorithm on two slightly dissimilar platforms.

In future, it is intended to further optimize the ground classification and tracking components and to replace the detector with more robust mechanism capable of performing object classification task. Moreover, it is envisioned to spare computational resources to incorporate visual object classification and utilize LiDAR-based spatial information for tracking.

## Figures and Tables

**Table 1:** Datasets information with tracking evaluation metrics.

Dataset	Frame Count	Objects	A	P	FP	FN	IDSW	MT (%)	PT (%)	ML (%)
0001	114	11	79.2	1.1	31	15	1	72.7	0.18	0.09
0002	83	2	88.37	0.4	0	5	0	100	0	0
0005	160	15	88.9	0.75	21	46	0	60	26.7	13.3
0009	453	79	75.8	1.7	187	261	4	57.5	18.7	25
0013	150	2	96.1	0.3	5	1	0	100	0	0
0017	120	4	96.7	1	4	0	0	100	0	0
0018	276	11	92.6	0.82	10	8	0	81.8	0	18.1
0048	28	5	86.5	1	7	8	0	71.4	14.3	14.2
0051	444	37	80.6	1.3	48	87	0	65	16	18.9
0057	367	13	93.14	0.72	37	6	0	100	0	0
	**2195**	**179**	**87.8%**	**<0.9m**	**8.2%**	**8.6%**	**5**	**80.8%**	**7.6%**	**9%**

The accumulated percentage of FP and FN are computed from the total measurements taken.

**Table 2:** Time consumption by main components of MODT on desktop and Jetson board.

Dataset	Objects	Ground Classifier (max. ms)	Clustering (max. ms)	Tracking (max. ms)	Total Time (max. ms)
0001	14	16.4/62.5	14.5/32.8	9.4/23	**40.3/118.3**
0002	3	15.4/65	14.5/33.3	3.4/7.3	**33.3/105.6**
0005	15	15.9/61.7	16.6/34.5	12/32.5	**44.5/128.7**
0009	95	15.9/62.8	15.7/34.9	15.9/60.3	**47.5/158**
0013	9	17/69	14.5/31.5	9.1/18.2	**40.6/118.7**
0017	4	15.4/72.4	12.7/28	2.5/6.6	**30.6/107**
0018	15	15.4/62.6	13.1/26.6	2.8/8.8	**31.3/98**
0048	7	15.1/59.5	15.9/35.2	9.1/19.2	**40.1/113.9**
0051	47	15/72	12.8/28	11.9/49.3	**39.7/149.3**
0057	26	15.3/62.3	12.8/27	7.1/20.5	**35.2/109.8**

The computation times on the desktop and Jetson board are separated by a forward slash.

**Table 3:** Comparison of tracking evaluation metrics and computation time.

Method	A	P	FN (%)	FP (%)	IDSW (%)	Computation Time ^1^ (ms)
Proposed Framework	87.8	<0.9	8.2	8.6	2.7	38.2
MODTUSU [6]	86.12	n/a	11.89	1.92	39	71.1
Tracking circle [9]	86.5	<0.2	3.5	8	0.9	86
Generative [8]	77.7	<0.14	8.5	10.1	3.6	n/a
Energy [10]	84.2	<0.12	5.8	2.77	n/a	n/a
BUTD [10]	89.1	<0.16	2.6	7.6	n/a	n/a

^1^ Average computation times on desktop computing environments are mentioned.

## Supplementary Materials

Supplementary File 1
